# Primary mesenteric neuroendocrine tumor: Case report

**DOI:** 10.1016/j.ijscr.2023.108517

**Published:** 2023-07-22

**Authors:** Alejandro González-Muñoz, Edgar Javier Aguirre-Salamanca, Natalia Andrea Rivera-Rincón, José Gabriel Rodríguez-Narvaez, Pablo González-Sierra, Camilo Ramírez-Giraldo

**Affiliations:** a Universidad del Rosario, School of medicine and Health Sciences, Bogotá D.C., Colombia; b Hospital Universitario Mayor Mederi, Bogotá, Colombia

**Keywords:** Mesenteric tumor, Neuroendocrine tumors, Primary mesenteric tumor

## Abstract

**Introduction and importance:**

Neuroendocrine tumors most frequently originate from the gastrointestinal tract (GIT). Their presentation in tissues other than the GIT and pancreas is usually due to metastatic involvement from lesions at these sites. There have been a few cases of neuroendocrine tumors identified in tissues such as the mesentery and peritoneum, without identification of a primary lesion supporting their origin as metastasis.

**Case presentation:**

We present the case of a patient with abdominal pain, in whom a primary mesenteric neuroendocrine tumor was identified. The patient completed one year of follow-up without identification of an additional lesion. Case Reported in line with the SCARE criteria.

**Clinical discussion:**

This is a rare condition with few reports in the literature, without significant changes in its classification or management.

**Conclusion:**

The search for a primary lesion and follow-up are essential to characterize the presence of primary mesenteric neuroendocrine tumors.

## Highlights

• Neuroendocrine tumors commonly originate from the gastrointestinal tract.
• There are few reports in the literature on primary neuroendocrine tumors of the mesentery.
• The follow-up of neuroendocrine tumors is done using chromogranin A, 5-HIAA, scintigraphy, and octreotide scan.
• The management of neuroendocrine tumors typically involves surgery, including in this presentation.

## 1 Introduction

Enterochromaffin cells or Kulchitsky cells are neuroendocrine cells primarily located in the epithelium of the gastrointestinal tract (GIT). They play a crucial role in various functions of the GIT, such as motility and secretion. Neuroendocrine tumors (NETs) predominantly arise from these cells and are mostly benign [1,2]. Neuroendocrine tumors (NETs) are so named due to the identification of dense core granules similar to those found in serotonergic neurons, and the endocrine component refers to the synthesis and secretion of monoamines. Given the widespread presence of neuroendocrine cells in the body, these types of neoplasms are described in the central nervous system, respiratory tract, larynx, gastrointestinal tract, thyroid, skin, breast, and urogenital system. The most common primary locations for NETs are the gastrointestinal tract (up to 67 % of cases) and lungs. However, they are rare tumors with an incidence of 5.86 per 100,000 inhabitants per year and a prevalence of 103,312 cases in the United States [3,4].

NETs are classified according to their location. For the gastrointestinal tract, they can be divided into Gastropancreatic NETs and Gastrointestinal NETs. In 2015, the National Comprehensive Cancer Network (NCCN) included tumor differentiation, mitotic rate, and Ki-67 in the classification of NETs. These factors determine the tumor grade, which guides treatment decisions [3].

Considering the origin of NETs, the occurrence of extra-GIT (extra gastrointestinal tract) lesions is infrequent, assuming the previously described information. In such cases, the presence of a primary tumor should be ruled out, as we seen in the mesenteric carcinoid tumors (usually metastatic). However, some reports in the literature describe the presence of extra-GIT lesions without identification of an alternative origin [5,6]. It is possible to associate the presence of NETs with multiple endocrine neoplasia type 1 (MEN1), an autosomal dominant syndrome, in which the possibility of presenting gastropancreatic NETs stands out [[2], [3], [4]].

The presence of chromogranin A and 5-hydroxyindoleacetic acid (5-HIAA) indicates the presence of NETs and is supplemented with imaging studies such as scintigraphy and octreotide scan to rule out metastasis and identify the primary tumor [1,2,[4], [5], [6]]. Here, we present a case report of a patient with a compressive mass in the mesenteric root, showing a well-differentiated NET without evidence of a primary tumor and negative serum biomarkers during one year of follow-up.

## 2 Case report

**Fig. 1:**
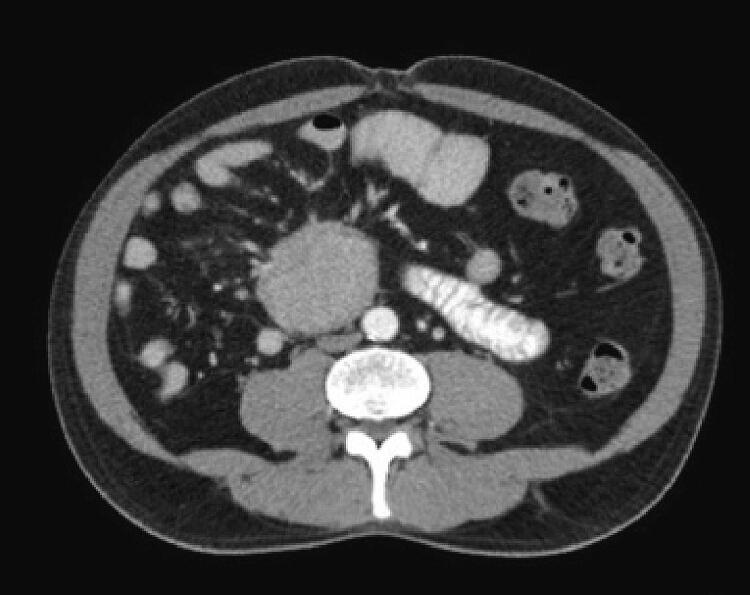
Axial contrast-enhanced abdominal CT scan section, showing a round, homogeneous, solid mass in the mesogastrium, with compressive effect on the inferior vena cava (IVC).

**Fig. 2:**
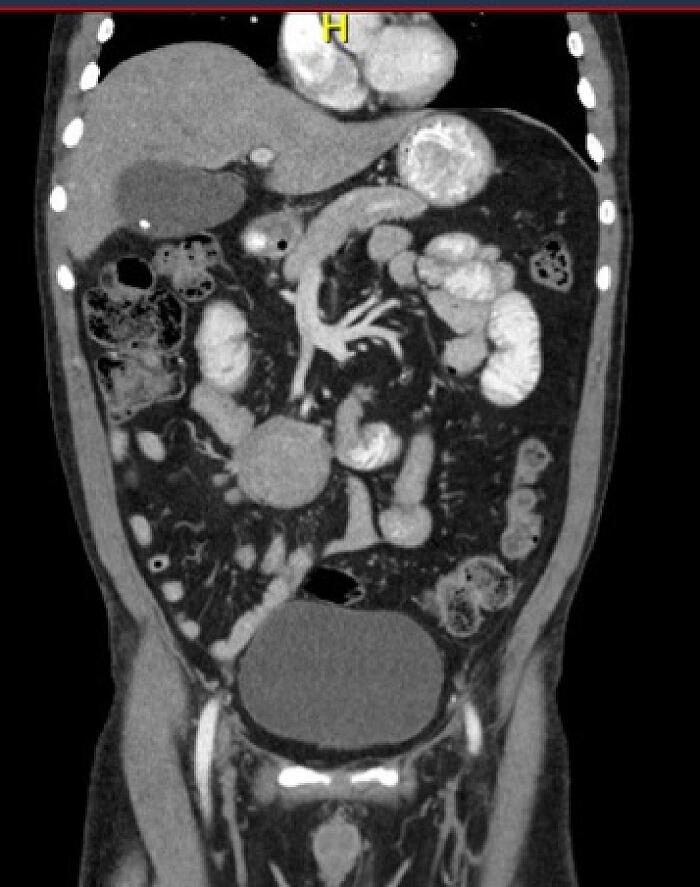
Coronal contrast-enhanced abdominal CT scan section, showing a round, homogeneous, solid mass in the mesogastrium, with compressive effect on the inferior vena cava (IVC).

A 56-year-old man with a history of appendectomy presented to the emergency department with acute abdominal pain in the right hypochondrium and epigastrium, associated with oral intolerance. Upon admission, there were no signs of peritoneal irritation, clinical signs of systemic inflammatory response, or positive inflammatory markers. Due to persistent symptoms, an abdominal ultrasound was performed, showing suggestive images of retroperitoneal lymph node involvement. Complementary contrast-enhanced abdominal CT showed a round, homogeneous, solid mass in the mesogastrium, causing compression on the inferior vena cava (IVC) and displacement of the second portion of the duodenum (Fig. 1, Fig. 2).

The patient underwent exploratory laparoscopy, identifying a mass of approximately 5 × 6 cm located at the root of the mesentery, in contact with the inferior vena cava (IVC), but with a cleavage plane between this structure. Vascular compromise of the small intestine was identified from 210 cm of the suspensory angle of the duodenum to 60 cm of the ileocecal valve, with impaired perfusion in this portion of the ileum. It was decided to convert to laparotomy, performing dissection using ultrasonic energy around the described mass. Due to direct involvement of the vasculature of the distal ileum, 60 cm of compromised small intestine were resected, and a lateral-lateral mechanical anastomosis was performed using the Barcelona technique. At the end of the procedure, the abdominal cavity was explored, and no evidence of lesions in the length of the small and large intestines, stomach, liver, pancreas, and intraperitoneal rectum was found.

The patient's hospital stay was prolonged due to postoperative ileus, initially managed with intravenous fluids and hydroelectrolyte replacement, however, a reintervention was required on the tenth day of management, intraoperatively identifying early adhesions. Adhesiolysis was performed without additional documentation of other alterations. Subsequently, the patient had a satisfactory postoperative recovery and was discharged on the fifth day after the reintervention.

**Fig. 3:**
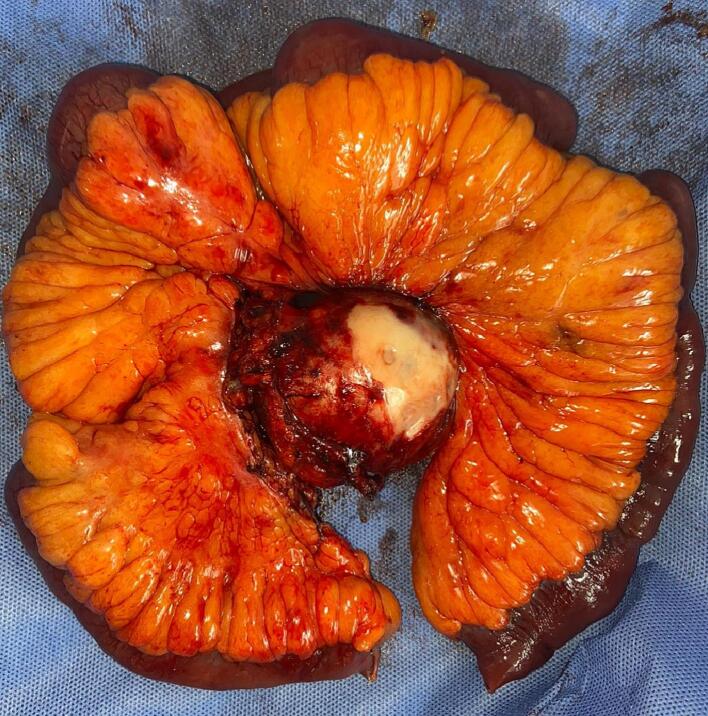
Macroscopic specimen of pathology, including 60 cm of small intestine with congested surface, adhered to the mesentery, with a large mass measuring 7 × 5 × 2 cm, congested, with fibrous and hemorrhagic areas; no involvement of the intestine by the mass is defined.

**Fig. 4:**
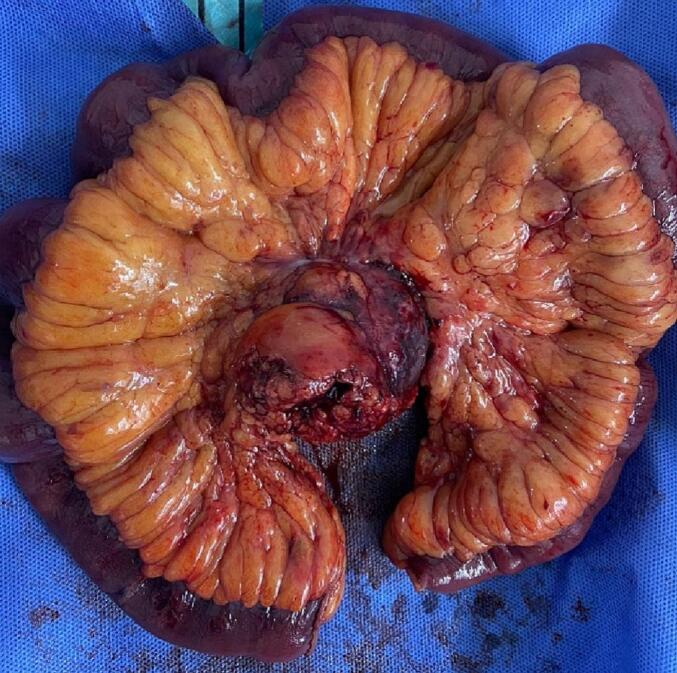
Macroscopic specimen of pathology, including 60 cm of small intestine with congested surface, adhered to the mesentery, with a large mass measuring 7 × 5 × 2 cm, congested, with fibrous and hemorrhagic areas; no involvement of the intestine by the mass is defined.

In pathology, a specimen was received including 60 cm of congested small intestine adhered to the mesentery, with a large congestive mass measuring 7 × 5 × 2 cm, with fibrous and hemorrhagic areas. No involvement of the intestine by the mass was defined (Fig. 3, Fig. 4). Microscopic findings indicated grade 1 well-differentiated neuroendocrine tumor (NET) with a mitotic count of one figure per 2 mm2 and a Ki-67 index of 1 %. Sixteen lymph nodes were identified, with one lymph node involved by the NET, and tumor-free margins were reported in the mesenteric tissue. There was no evidence of tumor necrosis, vascular invasion, or perineural invasion. Immunohistochemistry demonstrated positivity for cytokeratin AE1/AE3, chromogranin, synaptophysin, and CDX2, with negativity for TF-1, CK7, and CK20, consistent with the findings of a NET.

During the first six months of follow-up, the patient was evaluated by endocrinology and oncology, and the study was complemented with upper gastrointestinal endoscopy and colonoscopy. A small hyperplastic polyp was resected during the colonoscopy, which tested negative for malignancy. Negative results were also obtained for chromogranin A (90 ng/ml) and 5-HIAA (9 mg/24 h). Additionally, a technetium scintigraphy showed somatostatin receptor expression in the ascending and transverse colon, leading to direct confirmation with repeated endoscopic studies, which did not identify any additional lesions.

Based on the results of the scintigraphy, a PET-CT scan with gallium was performed, which showed no evidence of somatostatin receptor overexpression. Chromogranin and 5-HIAA controls at ten months of follow-up remained negative, and the patient is currently asymptomatic.

## 3 Discussion

Primary mesenteric tumors are a rare entity, with few reports available in the literature [1,2,[4], [5], [6]]. The majority of primary mesenteric tumors tend to have a benign behavior, including varieties such as desmoid tumors, lipomas, Schwannomas, paragangliomas, and less frequently, neuroendocrine tumors (NETs) and sarcomas, which necessitates ruling out their origin as metastases [1]. Neuroendocrine tumors arise from enterochromaffin cells present in multiple organs and can be characterized by the secretion of bioactive substances into the bloodstream [6]. The majority (over 90 %) of gastrointestinal NETs are usually located in the appendix, small intestine, and rectum [2]. The approximate incidence of NETs is around 3.56 per 100,000 individuals, with a median age of 64 years [5]. A female predominance has been documented (2.5:1), with a prevalence of 103,312 cases in the United States [3].

Radiological differential diagnoses for hypervascular extramural subepithelial lesions include Castleman's disease, metastatic disease, lymphadenopathies, and neuroendocrine tumors [1]. On computed tomography (CT), mesenteric NETs often present with variable degrees of fibrosis, calcification, and focal or diffuse neurovascular invasion [2]. Serum chromogranin A levels are often elevated in these patients, with a sensitivity and specificity ranging from 70 % to 100 %. In our case, since there was no initial suspicion of a NET, no initial reference serum markers were available, but postoperatively, negative markers were observed.

Neuroendocrine tumors, previously referred to as carcinoids, are more frequently generated in the gastrointestinal tract; Histopathological evaluation typically reveals structural patterns such as nests, cords, and ribbons, with strong expression of neuroendocrine markers like synaptophysin and chromogranin A, which are shared with paragangliomas, excluding this diagnosis based on cytokeratin positivity [1,6].

These tumors often present with symptoms of intestinal obstruction, abdominal pain, or a mass sensation, and in other cases, they may be associated with carcinoid syndrome, which is absent in non-secreting tumors [5]. Carcinoid syndrome can be evaluated by measuring chromogranin A or determining urinary excretion of 5-HIAA [6]. Metastatic presentations have been documented in these neoplasms before reaching 1 cm in diameter, occurring in up to 30 % of cases without detectable primary tumors. Additionally, 80 % of patients often present with nodal involvement at the time of diagnosis [4].

The World Health Organization introduced a classification system for NETs based on their proliferative activity determined by mitotic rate and the Ki-67 index (Table 1). Low-grade tumors have a better prognosis [6].

**Table 1:** Classification of neuroendocrine tumors according to the WHO.

Grades	Mitotic rate	Ki67
1	<2/10 high power fields	<3 %
2	2–20/10 HPF	3–20 %
3	>20/10 HPF	>20 %

When identifying a localized NET in the mesentery, it is always important to rule out metastatic involvement, as it is documented in 40–80 % of gastrointestinal NETs [1,2]. Complementary studies such as tomographic, endoscopic, scintigraphy, octreotide scan, and surgical exploration are essential to rule out metastatic involvement.

Surgical resection is usually the cornerstone of management for carcinoid tumors, including the resection of surrounding lymph nodes [2,5], as performed in the presented case, achieving complete resection of the lesion.

Similar cases were found in the literature. Lee et al. [1] reported the case of a 54-year-old female patient with a well-defined hypervascularized mass at the gastrohepatic ligament, who underwent laparoscopic resection, achieving complete excision of the mass, with no additional lesions identified in the abdominal cavity, resulting in a diagnosis of primary mesenteric NET. Kim [2] reported a case in 2014 of a patient who presented with a mass sensation. Abdominal computed tomography revealed a mass of approximately 15 × 10 cm arising from the mesentery of the transverse colon, and histopathological examination confirmed a grade 3 NET. Agarwal et al. [5] reported another case in a female patient with documentation of a 3.8 × 2.8 cm mass at the root of the mesentery and a concurrent hepatic lesion. Both lesions were resected, confirming a grade 1 NET. This case report was written following the SCARE criteria [7].

## 4 Conclusion

There are few reported cases of peritoneal NETs in the available literature. In the present reported case, after one year of follow-up, there is no evidence of a primary lesion to determine its metastatic origin. This case adds to the previously described cases, which amount to no more than ten cases in the available literature. Metastasis of the lesion cannot be entirely ruled out.

According to the reviewed literature, the identification of extra-gastrointestinal and pancreatic NETs warrants an exhaustive search for a primary tumor in these locations, as was done in the present case. However, no additional lesions have been identified at the moment, leaving the possibility of identifying these extra-GIT as a rare occurrence, as in the present case.

## CRediT authorship contribution statement

AGM: Data collection, photography, literature review, writing.

EJA, NAR, CRG: Thematic consultation, writing.

JGR, PGS: Literature review, writing.

## Consent

Informed consent was obtained from the patient, which was accepted by the ethics committee of the Hospital Universitario Mayor Mederi, Bogotá, Colombia.

## Ethic statement

Ethical approval for this study (DVO005 2315 CV21696) was provided by the ethical committee of the Hospital Universitario Mayor Mederi, and Rosario University, Bogotá, Colombia (06/05/23).

## Declaration of competing interest

None of the authors have any conflicts of interest.
